# Surgical treatment of tumor-induced osteomalacia: a retrospective review of 40 cases with extremity tumors

**DOI:** 10.1186/s12891-015-0496-3

**Published:** 2015-02-26

**Authors:** Zhi-jian Sun, Jin Jin, Gui-xing Qiu, Peng Gao, Yong Liu

**Affiliations:** Department of Orthopedics, Peking Union Medical College Hospital, Chinese Academy of Medical Sciences and Peking Union Medical College, Dongcheng District Shuaifuyuan No.1, Beijing, 100730 China

**Keywords:** Tumor-induced osteomalacia, Surgical treatment, Extremity, Tumor resection

## Abstract

**Background:**

Tumor-induced osteomalacia (TIO) is a rare syndrome typically caused by mesenchymal tumors. It has been shown that complete tumor resection may be curative. However, to our knowledge, there has been no report of a large cohort to exam different surgical approaches. This study was aimed to assess outcomes of different surgical options of patients with tumor-induced osteomalacia at a single institution.

**Methods:**

Patients with extremity tumors treated in our hospital from January, 2004 to July, 2012 were identified. The minimum follow-up period was 12 months. Patient’s demography, tumor location, preoperative preparation, type of surgeries were summarized, and clinical outcomes were recorded. Successful treatment was defined as significant symptom improvement, normal serum phosphorus and significant improvement or normalization of bone mineral density at the last follow-up. Differences between patients with soft tissue tumors and bone tumors were compared.

**Results:**

There were 40 (24 male and 16 female) patients identified, with an average age of 44 years. The tumors were isolated in either soft tissue (25 patients) or bone (12 patients) and combined soft tissue and bone invasion was observed in 3 patients. For the primary surgery, tumor resection and tumor curettage were performed. After initial surgical treatment, six patients then received a second surgery. Four patients were found to have malignant tumors base on histopathology. With a minimum follow-up period of 12 months, 80% of patients (32/40) were treated successfully, including 50% of patients (2/4) with malignant tumors. Compared to patients with bone tumor, surgical results were better in patient with soft tissue tumor.

**Conclusions:**

Surgical treatment was an effective way for TIO. Other than tumor curettage surgery, tumor resection is the preferred options for these tumors.

## Background

Tumor-induced osteomalacia (TIO), also known as oncogenic osteomalacia, is a rare syndrome characterized by hypophosphatemia, hyperphosphaturia, reduced 1,25-dihydroxyvitamin D concentration, and osteomalacia caused by typical benign mesenchymal tumors [1,2]. The pathogenic tumor secretes so-called phosphatonins [3], such as fibroblast growth factor 23 (FGF23) [4], frizzled related protein-4 [5], matrix extracellular phosphoglycoprotein [6] and FGF7 [7], which causes reduced reabsorption of phosphate in the proximal renal tubule. To date, FGF23 is thought to be the primary clinically relevant phosphatonin [8].

Locating the tumor is often challenging as it is often small in size and may be widely distributed. Functional imaging, including octreotide scintigraphy [9] and positron emission tomography along with computed tomography (PET/CT) [10], anatomical images, including magnetic resonance imaging (MRI), computed tomography (CT) and ultrasonography (USG), and venous sampling [10,11] have been used. Once the tumor is successfully localized, surgical removal of the tumor should be planned.

To our knowledge, no study has examined the efficacy of different surgical approaches of TIO in a large cohort perhaps due to rarity and wide distribution of the tumors. Depending on the anatomical location of the tumor, surgery is often performed by different specialties, including orthopedists, otorhinolaryngologists, dentists, urologists and thoracic surgeons [12,13]. Owing to the anatomical features of the tumor, preoperative preparation and choices of surgical approaches are also different. We reported our experience of bone and/or soft tissue tumors of the extremities in our institution. The primary aim of this study was to evaluate the overall outcomes of different surgical treatment and to determine the preferred surgical options. The second aim was to compare the surgical outcomes between soft tissue tumor and bone tumor.

## Methods

### Patients

We performed a retrospective review of TIO patients with bone and/or soft tissue tumors located at either upper or lower extremities. Patients with tumors in the maxillofacial region or visceral organs were not included in current study. From January, 2004 to July, 2012, 40 TIO patients with extremity tumors and treated surgically at our hospital were identified. Patients’ medical records, surgical procedures including preoperative preparations, and pathology reports were reviewed. This study was approved by the institutional review board of Peking Union Medical College Hospital. And written informed consent was obtained from all participants in the study.

### Preoperative preparation

All patients suspected of TIO were referred to our Orthopedic department from the Endocrinology department. Multiple imaging modalities were used to identify tumor, including functional studies (octreotide scintigraphy and/or PET/CT) and anatomic studies (CT, USG and/or MRI) [12].

### Operative procedure

Tumor biopsy was not performed before removal of the tumor for the following reasons: (1) the tumor is usually small and is not biopsiable prior to excision; (2) the tumor is typically characterized as phosphaturic mesenchymal tumor (PMT) and malignant tumors are rarely seen. If pathology after surgery suggested a malignant tumor, a secondary operation with extensive margins would be offered.

For soft tissue tumor, complete tumor, including the integrated capsule, was resected. If the tumor was close to bone margin and bone involvement could not be excluded, part of the cortical bone was also removed.

**Figure 1:**
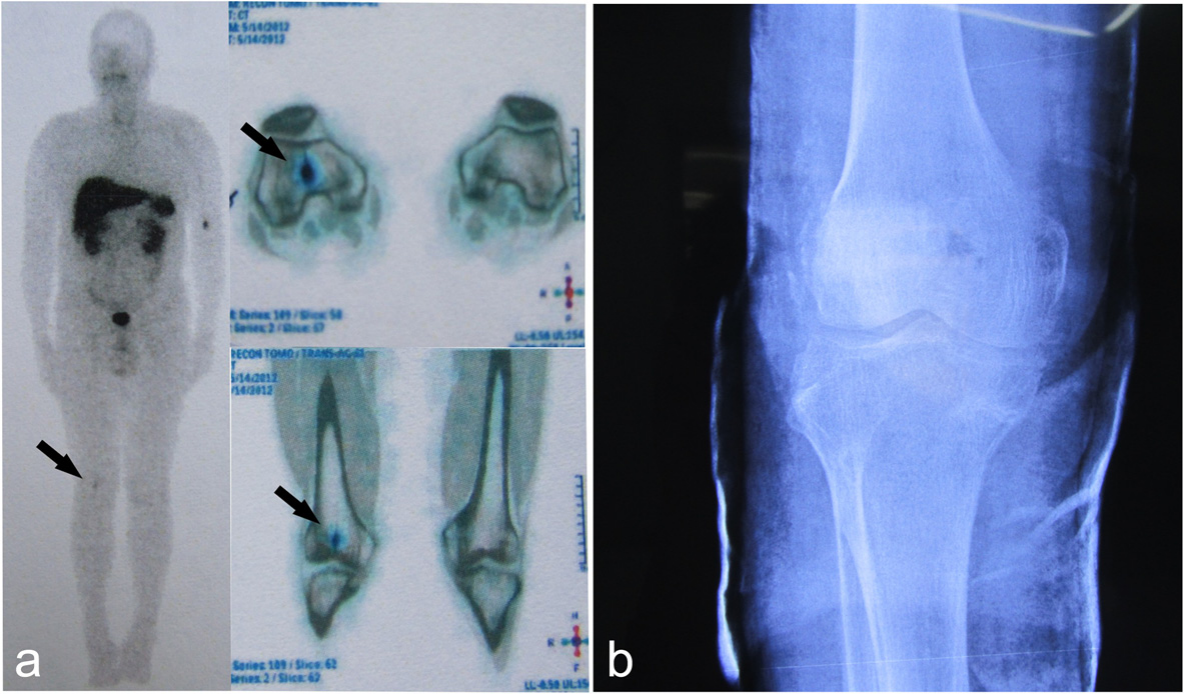
**A 63 year-old male patient complained bone pain and fatigue for five year.** Decreased serum phosphorus (0.39 mmol/L) and 1, 25-dihydroxyvitamin D (14.4 pg/mL) was observed, and 24-hour urine phosphate and TMP/GRF was 19.5 mg and 0.56 mg/dL, respectively. **(a)** ^99^Tc^m^-OCT showed high expression of somatostatin receptor in his right knee (black arrow). **(b)** Curettage and bone allograft were performed and serum phosphorus returned to normal eight days after surgery. Clinical symptoms were significantly improved at one year follow-up.

If the tumor was located in skeletal tissue, extensive tumor curettage and bone allograft was usually performed at the initial surgery with curettage extending for at least 5 mm from the presumed tumor margin according to MRI due to concern about surgical morbidity with complete resection. Yet it should be done cautiously to not breach the cortical bone. A typical case was given in Figure 1. If the tumor was not completely removed in first surgery, tumor resection and custom-made artificial prosthesis replacement was offered. With this surgical approach, the complete tumor with at least 20 mm margins would be resected. For patients not suitable for prosthesis replacement or for those who refused, a second curettage and bone allograft surgery was attempted. In cases where tumor curettage was difficult, tumor resection and prosthesis replacement would be performed as the first option. The resection range would include at least 20 mm normal tissues to the tumor margin. For the tumor infiltrating both soft tissue and bone, extensive tumor resection and curettage was performed.

If the histopathology after surgery suggested the tumor was malignant, extensive tumor resection with more normal tissues to the tumor margin was suggested. For patients with extensive tumor involvement, amputation was recommended.

### Postoperative monitoring and follow-up

Serum phosphorus was routinely tested 2 hours, 6 hours, 12 hours, 24 hours, 48 hours, 72 hours, 5 days, and 7 days after surgery, and then every 2–3 days until serum phosphorus levels became normal or patients were discharged. FGF-23 was not measured because we lacked the capability to routinely do so.

For the patients whose pathological result indicated a malignant tumor, a second surgery would be suggested and local radiotherapy was scheduled as necessary.

All the patients were asked to follow-up 3 months after surgery at orthopedic and endocrine clinics, and then every 6–12 months. Secum phosphorus, 1, 25-dihydroxyvitamin D and bone mineral density (evaluated by dual-energy X-ray bone density screening) were regularly monitored during the follow-up period. For the patient whose serum phosphorous didn’t return to normal, medical treatment would be arranged by endocrinology physicians. Successful treatment was defined as significant symptom improvement, normal serum phosphorus and significant improvement or normalization of bone mineral density at the last follow-up.

### Statistical analysis

Data were analyzed using statistical software SPSS version 16.0 (SPSS Inc, Chicago, IL). Fisher’s exact test was used to test the difference of gender ratio, pathological fracture rate, secondary operation rate and overall serum phosphorus recovery rate between patients with soft tissue tumors and bone tumors. Independent t-test was used to test the difference of age, disease course, serum phosphorus level, tumor size, operating time, bleeding volume and follow-up period between patients with soft tissue tumors and bone tumors. Statistically significant differences were defined by *p* < 0.05.

## Result

### General characteristics of TIO patients

A total of 40 patients (24 males and 16 females) with an average age of 44 ± 12 years (range of 20–67 years) were identified in this cohort. All patients had symptoms of bone pain, fatigue and difficulty walking. 75% (30/40) of patients had at least one site of pathological fracture caused by osteomalacia, primarily in ribs and lower extremities. Decreased serum phosphorus was seen in all patients with an average of 0.44 ± 0.12 mmol/L (range of 0.27-0.66 mmol/L) (normal range of 0.81-1.45 mmol/L), whereas serum calcium was normal in all patients with an average of 2.31 ± 0.12 mmol/L (range of 2.10-2.62 mmol/L). Alkaline phosphatase (ALP) was usually elevated with an average of 277 ± 126U/L (range of 66-759U/L). The average 1,25-dihydroxyvitamin D was 18.7 ± 11.8 pg/mL (range of 7.2-45.8 pg/mL). 24-hour urine phosphate and 24-hour urine calcium was 743 ± 468 mg (range of 67-2171 mg) and 103 ± 68 mg (range of 1.2-281 mg), respectively. And the average TMP/GFR was 0.39 ± 0.2 mg/dL (range of 0.12-0.99 mg/dL).

All 40 patients received ^99^Tc^m^-OCT (^99^Tc^m^-octreotide) and 39 showed high expressions at different regions. The tumor of the patient with negative ^99^Tc^m^-OCT (see Table 1, patient 17) was discovered through physical examination. And the tumor was located at subcutaneous tissue of her right thigh. Six patients received PET/CT and all showed hypermetabolic zone the same as ^99^Tc^m^-OCT did. The tumors were located at soft tissue and bone in 25 and 12 patients, respectively. Both soft tissue and bone invasion was observed in three patients. 90% (36/40) of the tumors were in the lower extremities. The tumor location for each patient is listed in Table 1.

**Table 1:** **Surgical options and effects for the treatment of patients with TIO**

**No.**	**Gender/age (year)**	**Tumor location**	**Primary surgical procedure**	**Histopathology**	**SP level/recovery day**	**Secondary surgery/surgical procedure/SP level**
**1**	F/42	soft tissue of right thigh	Tumor resection	MPNST	Unnormalized	No
**2**	F/41	soft tissue of left medial ankle	Tumor resection	GCTTS	Normalized/8 days	No
**3**	F/53	Bone tissue of right tibial plateau	Curettage and bone allograft	PMT	Unnormalized	Yes/Tumor resection and prosthesis reconstruction/Normalized
**4**	M/52	Bone tissue of medial condyle of right femur	Curettage and bone allograft	No tumor cells	Unnormalized	No
**5**	M/41	Soft tissue of right thigh	Tumor resection	PMT	Normalized/6 days	No
**6**	M/30	Bone and soft tissue of left distal femur	Tumor resection and curettage with cement reconstruction	Malignant PMT	Unnormalized	No
**7**	F/28	Soft tissue of left thigh	Tumor resection	PMT	Normalized/7 days	No
**8**	M/29	Soft tissue of right foot	Tumor resection	PMT	Normalized/5 days	No
**9**	M/57	Soft tissue of left leg	Tumor resection	PMT	Normalized/6 days	No
**10**	F/47	Soft tissue of left thigh	Tumor resection	PMTMCT	Normalized/3 days	No
**11**	M/57	Soft tissue of right thigh	Tumor resection	PMT	Normalized/7 days	No
**12**	M/27	Bone tissue of left greater trochanter	Curettage and bone allograft	PMT	Unnormalized	Yes/Curettage and bone allograft/Unnormalized
**13**	F/33	Bone and soft tissue of right leg	Partial tumor resection	PMT	Unnormalized	Yes/Amputation/normalized (malignant PMT)
**14**	F/43	Soft tissue of left leg	Tumor resection and partial fibula resection	PMT	Normalized/3 days	No
**15**	F/34	Bone and soft tissue of right leg	Tumor resection and curettage with bone allograft	Malignant PMT	Unnormalized	Yes/Amputation/Normalized
**16**	M/43	Soft tissue of left forearm	Tumor resection and partial radius resection	PMT	Normalized/12 days	No
**17**	F/49	Soft tissue of right thigh	Tumor resection	PMT	Normalized/5 days	No
**18**	M/54	Soft tissue of right foot	Tumor resection	PMT	Normalized/7 days	No
19*	F/29	Soft tissue of right foot	Tumor resection	PMT	Unnormalized	No
**20**	M/39	Bone tissue of right femur head	Tumor resection and THA	PMT	Normalized/6 days	No
**21**	F/48	Soft tissue of left foot	Tumor resection	PMT	Normalized/3 days	No
**22**	M/66	Soft tissue of left thigh	Tumor resection	PMT	Normalized/2 days	No
**23**	M/50	Bone tissue of left tibial plateau	Curettage and bone allograft	PMT	Unnormalized	Yes/Tumor resection and prosthesis reconstruction/unnormalized
**24**	F/67	Soft tissue of left foot	Tumor resection	PMT	Normalized/3 days	No
**25**	M/47	Soft tissue of right hip	Tumor resection	PMT	Normalized/6 days	No
**26**	F/59	Bone tissue of medial condyle of right femur	Curettage and bone allograft	No tumor cells	Normalized/7 days	Yes/Tumor resection and prosthesis reconstruction/normalized (PMT)
**27**	M/37	Soft tissue of left foot	Tumor resection	PMT	Normalized/3 days	No
**28**	M/50	Soft tissue of right arm	Tumor resection	PMT	Normalized/7 days	No
**29**	M/29	Soft tissue of right leg	Tumor resection	PMT	Normalized/5 days	No
**30**	M/20	Bone tissue of right ulna	Tumor resection and bone allograft	PMT	Unnormalized	No
**31**	M/50	Bone tissue of right tibial plateau	Curettage and bone allograft	No tumor cells	Unnormalized	No
**32**	F/47	Bone tissue of right femur head	Tumor resection and THA	PMT	Normalized/6 days	No
**33**	M/32	Bone tissue of right greater trochanter	Curettage and bone allograft	PMT	Normalized/8 days	No
**34**	F/38	Bone tissue of right greater trochanter	Curettage and bone allograft	PMT	Normalized/7 days	No
**35**	M/39	Soft tissue of left thigh	Tumor resection	PMTMCT	Normalized/4 days	No
**36**	M/63	Bone tissue of right distal femur	Curettage and bone allograft	PMT	Normalized/8 days	No
**37**	M/46	Soft tissue of right foot	Tumor resection	PMTMCT	Normalized/2 days	No
**38**	M/31	Soft tissue of right wrist	Tumor resection	PMT	Normalized/5 days	No
**39**	F/57	Soft tissue of left thigh	No tumor was found	No tumor cells	Unnormalized	No
**40**	M/62	Soft tissue of right leg	Tumor resection	hemangioma	Normalized/7 days	No

SP, serum phosphorus; MPNST, malignant peripheral nerve sheath tumor; GCTTS, giant cell tumor of tendon sheath; PMT, phosphaturic mesenchymal tumor; PMTMCT, PMT mixed connective tissue variant; THA, total hip arthroplasty.

*This patient received tumor resection at the same region twice in other hospital and the tumor was not completely removed both times.

### Primary surgical treatment

All patients received tumor resection or curettage by the single senior doctor. 39 of 40 patients in this series were primary surgical cases (except patient 19). The mean operating time and mean bleeding volume was 103 ± 54 min (range of 20-260 min) and 127 ± 257 mL (range of 20-1200 mL), respectively.

For soft tissue tumors, tumor resection was performed. With the help of pre- and intra-operative USG location, the tumor could usually be successfully isolated despite its small size, with the exception of patient 39. In three patients (patient 9, 14 and 19), the tumors were close to the bone, so some cortical bone tissues were removed together.

For bone tumors, tumor curettage and bone allograft was performed in nine patients; tumor resection and bone allograft was performed in one patient (patient 30); tumor resection and total hip arthroplasty (THA) was performed in two patients (patient 20 and 32).

For the three patients with both soft tissue and bone tumors, tumor resection and curettage was attempted first. In patient 13, the tumor was too widely involved, thus only part of it was removed.

### Secondary surgical treatment

10 patients required secondary surgery, including four patients with malignant tumors (patient 1, 6, 13 and 15) and six patients with bone tumors (patient 3, 4, 12, 23, 26 and 31). However, only six patients whose tumors were not completely removed received secondary surgery. Two patients (patient 3 and 23) with proximal tibial tumor received tumor resection and prosthesis reconstruction. One patient (patient 26) with distal femur tumor received tumor resection of distal femur and prosthesis reconstruction. One patient (patient 12) with greater trochanter tumor underwent tumor curettage and bone allograft again. The last two patients (patient 13 and 15) were diagnosed with malignant tumor and underwent amputation. Other patients refused secondary surgeries.

### Histopathology

Histopathology showed PMT in 34 patients (85%, 34/40), of which three patients showed malignant PMT [14]. Typical PMT are composed of spindled cells with a highly vascular, embedded in a distinctive myxoid to myxochondroid matrix, with “grungy” or flocculent calcification. Osteoclast-like giant cells, cicrocysts, prominent blood vessels, cartilage-like matrix and woven bone were admixed with these small cells. Spindled cells of benign PMT were of low nuclear grade and low mitotic activity. For malignant PMT, spindled cells were characterized as high nuclear grade, high cellularity and elevated mitotic activity. Involvements of surrounding soft tissue and cancellous bone were observed in these three patients with malignant PMT. Other histopathologic diagnoses included one malignant peripheral nerve sheath tumor (MPNST), one giant cell tumor of tendon sheath (GCTTS) and one hemangioma. No tumor tissues were found in three patients. The pathological results of each patient are listed in Table 1.

### Surgical results and follow-up

All patients were followed up for at least 12 months (average: 43 months; range: 12–106 months). Serum phosphorus returned to normal in 72.5% (29/40) of patients 5.6 ± 2.2 days (range of 2–12 days) after primary surgery. After secondary surgery, serum phosphorus became normal in three more patients. During a minimum 12 months follow-up period, significant clinical symptom improvements and improvements of bone mineral density were observed in all these patients. Thus successful treatment was achieved in 80% of patients (32/40). Eight patients still have decreased serum phosphorus and are receiving medical therapy. No patients died during follow-up period.

Four malignant tumors were found in this cohort. Two who received amputation achieved ensuing normal serum phosphorus (patient 13 and 15) and no tumor recurrence was observed at the last follow-up; the other two patients refused secondary operation and received irradiation with a median radiation dose of 50 Gy for five weeks, yet serum phosphorus did not become normal, despite that no distant metastasis was observed showed by ^99^Tc^m^-OCT (patient 1 and 6).

### Comparison of soft tissue tumor and bone tumor

Differences between patients with soft tissue tumors and bone tumors were further compared (Table 2). No statistical differences were found in gender ratio, age, disease course, pathological fracture rate, serum phosphorus level and tumor size. Although operating time of primary surgery was longer and bleeding volume was greater in patients with bone tumors, this was not statistically significant (*p* > 0.05). Secondary surgery rate was significantly higher in patients with bone tumors (*p* < 0.05). Although the overall serum phosphorus recovery rate in patients with soft tissue tumors was 88%, and only 58% in bone tumors, no statistical significance was found (*p* > 0.05). There was no difference in follow-up period, either.

**Table 2:** **Comparison of TIO patients with soft tissue tumor or bone tumor**

	**Soft tissue tumor (n = 25)**	**Bone tumor (n = 12)**
General characteristics		
Gender (F:M)	10:15	4:8
Age (year)	46 ± 12	44 ± 13
Disease course (year)	4.8 ± 2.3	7.7 ± 7.0
Pathological fracture (%)	32(8/25)	17(2/12)
SP level (mmol/L)^*^	0.44 ± 0.11	0.46 ± 0.15
Longest diameter of tumor cm)	2.9 ± 1.4	2.2 ± 1.6
Primary operation message		
Operating time (minute)	85 ± 42	109 ± 39
Bleeding volume (mL)	72 ± 138	153 ± 281
Secondary operation rate (%)^#^	0(0/25)	33(4/12)
Overall SP recovery rate (%)	88(22/25)	58(7/12)
Follow-up period (month)	35 ± 31	26 ± 34

SP, serum phosphorus.

^*^The normal range of SP level was 0.81-1.45 mmol/L.

^#^ *p* < 0.05.

## Discussion

80% (32/40) of our study cohort was treated successfully through complete tumor removal surgery during an average follow-up period of 43 months, including 29 patients after primary surgery and three after secondary surgery. To gain complete remission of clinical symptoms, the tumor must be completely removed. For the eight patients in this cohort whose serum phosphorus levels were only partially normalized, their tumors were incompletely resected.

Soft tissue tumors usually had clear boundaries and were well-encapsulated, so complete tumor resection was straightforward. However, some tumors grew close to bone, so sometimes it was difficult to distinguish whether bone involvement existed; in these cases, some bone tissue was removed. In fact, some cortical bone was resected in three patients with soft tumors in this series, and all of them completely recovered. The prognosis for TIO patients with soft tissue tumor was good, as 88% of patients achieved normal serum phosphorus in this study. Of the three patients whose serum phosphorus did not return to normal, one had a malignant tumor (patient 1), one had two previous surgeries at the same site (patient 19), and one had an undetectable tumor (patient 39).

Surgical treatment of bone tumors seemed to be much more difficult, because it was often impossible to distinguish tumors with normal tissues during surgery. Preoperative preparation to determine curettage range was important and tumor curettage with at least 5 mm from tumor margins was suggested. However, only four of nine patients were successfully treated after primary tumor curettage. Harbeck et al. [15] reported a 34-year-old male patient with TIO, whose tumor was located to the left greater trochanter. Intraoperatively, a hand-held gamma probe after administration of ^111^Indium pentetreotide 1 day before surgery clearly identified the tumor. This report provided us another way to identify the tumor during surgery.

Bone tumor resection appeared to be more effective than curettage. In our study, two patients with femoral head tumors received total femur head resection and THA and both achieved successful treatment at the last follow-up. In addition, three patients with failed primary tumor curettage surgeries received secondary tumor resection and prosthesis reconstruction, two of which were successfully treated. Nevertheless, these kinds of surgeries are often associated with greater morbidity with loss of some limb function. Prosthesis related problems cannot be ignored either. Less invasive treatment has been reported in the literature. Hesse et al. [16] reported a 40-year-old woman with a tumor in her right femoral head. To preserve her hip joint, CT-guided radiofrequency ablation was performed for two rounds. Complete ablation was achieved, and no tumor recurrence was observed at one year follow-up. Tutton et al. [17] reported another patient with a tumor in his right iliac bone. CT-guided percutaneous ethanol ablation and percutaneous cryoablation was performed. The tumor was completely destroyed during 12 months follow-up. Although these results are promising, larger sample sizes are still needed. These are alternative methods and could be considered for patients who refuse surgical treatment.

Compared with widely tumor curettage surgery, tumor resection surgery seemed to be a better choice for TIO. However, considering anatomical and functional factors, it’s difficult to perform tumor resection in all cases as function could be severely compromised. Tumor curettage could be considered in cases where surgical morbidity was a concern and that attempting to curettage beyond the expected tumor margin was recommended.

The mesenchymal tumor was typically benign whereas malignant tumor was seen as well [2,12,14,18-21]. Four malignant tumors were identified in our series of cases. Of them, two received amputations, one received tumor resection and the last one received tumor curettage. For the last two patients, radiotherapy was arranged even though only a little data had suggested it to be strongly effective [19]. In fact, it was difficult to predict the behavior of these tumors only based on the morphologic findings [2,18]. In our cohort, two of three tumors involving both soft tissue and bone were proved to be malignant, which might be another characteristic to remind us of the behavior of these tumors.

There were some limitations for this study. Firstly, MRI or USG was not routinely performed during the follow-up time for patients with unnormalized serum phosphorus; instead, serum phosphorus and 1, 25-dihydroxyvitamin D as well as bone mineral density were regularly monitored. Only for those with decreased serum phosphorus and intending for further surgical treatment, residual tumors were examined. In addition, the minimum follow-up time was only 12 months, thus long term results could not be concluded. At last, surgeries were not always performed as surgeons planned. Some patients refused further or more invasive operations, which might influence the overall efficacy assessment of surgical treatment.

## Conclusions

We report the surgical management of 40 cases of TIO patients with extremity tumors, which is a relatively large cohort for this rare disease. The majority of patients were treated successfully after tumor removal surgeries. Complete tumor resection was the more effective surgical approach. When this was not feasible, tumor curettage with wide curettage margins could be considered, though persistent or recurrent disease might be more likely.

## Abbreviations

TIO: Tumor-induced osteomalacia
FGF23: Fibroblast growth factor 23
PET/CT: Positron emission tomography along with computed tomography
MRI: Magnetic resonance imaging
CT: Computed tomography
USG: Ultrasonography
PMT: Phosphaturic mesenchymal tumor
PMTMCT: PMT mixed connective tissue variant
ALP: Alkaline phosphatase
THA: Total hip arthroplasty
MPNST: Malignant peripheral nerve sheath tumor
GCTTS: Giant cell tumor of tendon sheath
PE: Physical examination
OS: Octreotide scintigraphy
SP: Serum phosphorus
